# Salvage of cardiopulmonary collapse caused by ethanol sclerotherapy for vascular malformations: clinical experience at a single center and literature review

**DOI:** 10.3389/fcvm.2024.1439839

**Published:** 2024-11-20

**Authors:** Lixin Su, Yan Zhao, Yuchen Shen, Xindong Fan, Zhenfeng Wang, Deming Wang, Qingyang Li

**Affiliations:** ^1^ Department of Vascular Anomaly, Fengcheng Hospital of Fengxian District, Shanghai, China; ^2^ Department of Interventional Therapy, Multidisciplinary Team of Vascular Anomalies, Shanghai Ninth People’s Hospital, Shanghai Jiao Tong University, Shanghai, China; ^3^ Department of Anesthesiology, Fengcheng Hospital of Fengxian District, Shanghai, China

**Keywords:** cardiopulmonary collapse, ethanol, vascular malformations, sclerotherapy, emergency

## Abstract

**Objective:**

This review aims to summarize the salvage experience of cardiopulmonary collapse occurring as a result of absolute ethanol sclerotherapy for vascular malformations.

**Methods:**

In total, we reviewed three cases of cardiopulmonary collapse induced by ethanol sclerotherapy for vascular malformations and described the details of the salvage procedure. Saturation of pulse oxygen (SpO_2_), end-tidal CO_2_, and invasive arterial pressure were the routine monitors for ethanol injection patients. Cardiopulmonary resuscitation, epinephrine, norepinephrine, and deoxyepinephrine were mainly used to correct circulation parameters. Manually ventilated via endotracheal intubation with 100% O_2_, increased respiratory rate were mainly used to correct Respiratory parameters.

**Results:**

All three cases were successfully salvaged without major complications. When cardiopulmonary collapse occurred, manual ventilation via endotracheal intubation with 100% O_2_, increased ventilation frequency and external cardiac compression were the emergency treatments. Epinephrine, norepinephrine, deoxyepinephrine infusion solely or combined were crucial to maintaining the basic vital signs.

**Conclusion:**

Despite the severity of cardiopulmonary collapse caused by ethanol sclerotherapy, it can be detected by close observation and reversed with timely treatment.

## Introduction

Vascular malformations are clinically problematic and often difficult lesions to treat. They display unique heterogeneous manifestations and can occur anywhere in the body. In the past decades, embolotherapy has been a mainstay of treatment, with many clinical studies demonstrating its varying degrees of efficacy. Nonetheless, although methods and materials for sclerotherapy are extensively varied and heavily studied, there is no clear consensus on the suitable embolic agents for various malformations (1–3). Yakes et al. first described the use of ethanol in malformation sclerotherapy in 1986 (4). Since then, ethanol has been increasingly used in high flows (AVMs) and lower flow venous, capillary, and lymphatic malformations (5).

Despite its high efficacy, ethanol is an extremely dangerous intravascular sclerotherapy agent, causing significant complications if it enters the systemic circulation. Ethanol sclerotherapy induces minor local complications including skin blistering, ulcerations, scar formation, and local nerve damage. Moreover, it may cause cardiopulmo­nary complications such as pulmonary embolism, pulmonary hypertension, and cardiac arrhythmias that potentially trigger car­diovascular collapse or even death (6–9). Thus, radiologists and anesthetists must be aware of the severity potential of these cardiopulmonary complications triggered by ethanol sclerotherapy, hence must be prepared to manage. Herein, we review three cases of cardiopulmonary collapse induced by ethanol sclerotherapy for vascular malformations and its periprocedural management.

## Patients

The present work was approved by the Institutional Review Board of Shanghai Ninth People's Hospital, Shanghai Jiaotong University School of Medicine [No. SH9H-2019-T309-2]; All procedures performed in studies involving human participants were in accordance with the ethical standards of the institutional with the 1964 Helsinki declaration and its later amendments or comparable ethical standards. Informed consent was obtained from all individual participants included in the study. Consent for publication was obtained for every individual person's data included in the study.

## Case 1

A 32-year-old female patient with a height of 166 cm and weight of 58 kg was admitted to undergo ethanol sclerotherapy for a large venous malformation in the left waist. All preoperative examinations, laboratory tests, electrocardiography, and chest x-ray were nor­mal. General anesthesia was intravenously induced using a bolus in­jection of 120 mg propofol and 40 mg rocuronium. Endotracheal intubation was inserted without difficulty and the patient was maintained using machine-controlled ventilation (tidal volume 300 ml, ventilation frequency 16 times per minute) on 68% nitrous oxide in oxygen with isoflurane at end-tidal concentrations of 1%–1.5%. Also, she was intravenously administered with 8 mg of atracurium benzenesulfonate. We applied routine patient monitors, including pulse oxygen saturation (SpO_2_), three-lead electrocardiogram (ECG), end-tidal CO_2,_ and invasive arterial pressure. After venography from direct puncture, absolute ethanol injection was routinely performed (10). Immediately after ethanol injection, the blood pressure and heart rate of the patient slightly increased to 135/90 mmHg and 90 beats/min, respectively. Thereafter, blood pressure and heart rate became stable throughout the proce­dure. A 22 ml dose of 99.7% ethanol was injected into the lesion for over 15 min. Consequently, we noted an abrupt decrease in her invasive arterial pressure; 50–55 mmHg systolic and 20–22 mmHg diastolic blood pressure with a simultaneous decrease of heart rate to 35–38 beats/min; end-tidal CO_2_ decrease to 19–21 mmHg; SpO_2_ decrease to 55%. Within 60 s of onset, peripheral pulses were lost and invasive arterial pressure failed to yield a measurement. The ECG (lead II) showed that the heart rate first becomes slightly fast and then slows down, followed by severe sinus bradycardia with escape rhythm, and finally ventricular fibrillation until cardiac arrest. The anesthetic agents were discontinued then the oxygen concentration in ventilation was increased to under 100% and manually ventilated via endotracheal intubation 30 times per minute. External cardiac compression was started with resultant palpable femoral pulsation. Epinephrine 1 mg was given intravenously and chest compression continued for 3 min before the pulses were restored. Blood pressure increased to 50/30 mmHg, SpO_2_ to 65%, heart rate to 55 beats/min. Subsequently, norepinephrine infusion was administered at 20 μg/2 min and repeated twice; as a result, blood pressure increased to 75/45 mmHg; SpO_2_ to 86%; heart rate to 135 beats/min. Deoxyepinephrine infusion was administered at 10 μg/2 min and repeated twice; consequently, blood pressure increased to 93/55 mmHg; SpO_2_ to 96%; heart rate decreased to 82 beats/min. After 50 min in the operating theater, the patient regained consciousness, began to spontaneously breathe, and obeyed commands. After removing the endotracheal tube, the patient was transferred to the intensive care unit. Echocardiography follow-up during the next day showed a normal heart with efficient biventricular function. After 3 days of observation, the patient completely recovered and was discharged.

## Case 2

A 28-year-old male patient with a height of 175 cm and weight of 68 kg was admitted to undergo ethanol sclerotherapy for venous malformation in the left back. Preoperative examinations, including endotracheal intubation, general anesthesia, absolute ethanol injection was routinely performed.

The cardiopulmonary collapse occurred in a total dose of 25 ml of 99.7% ethanol was injected into the lesion over 20 min. We observed an abrupt decrease in her invasive arterial pressure, 50–60 mmHg systolic and 18–20 mmHg diastolic blood pressure with a simultaneous decrease in her heart rate to 22–36 beats/min, end-tidal CO_2_ decrease to 20–23 mmHg, and SpO_2_ decrease to 56%. The anesthetic agents were discontinued then under and manually ventilated via endotracheal intubation with 100% O_2_ at 30 times per minute. Norepinephrine infusion was administered at 20 μg/2 min and repeated twice; consequently, the blood pressure increased to 94/65 mmHg, SpO_2_ to 92%, and heart rate to 125 beats/min. Then, deoxyepinephrine infusion was administered at 10 μg/2 min and repeated once; consequently, blood pressure increased to 105/68 mmHg, SpO_2_ to 98%, while heart rate decreased to 82 beats/min. After 35 min in the operating theater, the patient regained consciousness, began to spontaneously breathe, and obeyed commands. The endotracheal tube was removed and the patient completely recovered.

## Case 3

A 12-year-old female patient with a height of 152 cm and weight of 46 kg was admitted to undergo ethanol sclerotherapy for venous malformation in the lateral chest. Endotracheal intubation general anesthesia and absolute ethanol injection were routinely performed.

The cardiopulmonary collapse occurred in a total dose of 20 ml of 99.7% ethanol was injected into the lesion over 18 min. We observed an abrupt decrease in her invasive arterial pressure, 45–52 mmHg systolic and 16–20 mmHg diastolic blood pressure with a simultaneous decrease in her heart rate to 25–35 beats/min, end-tidal CO_2_ decrease to 20–22 mmHg, and SpO_2_ decrease to 55%. The anesthetic agents were discontinued then the patient was manually ventilated via endotracheal intubation with 100% O_2_, 25 times per minute. Norepinephrine infusion was administered at 6 μg; consequently, blood pressure increased to 100/70 mmHg, SpO_2_ to 91%, heart rate to 145 beats/min. Deoxyepinephrine infusion was administered at 6 μg/2 min and repeated once; consequently, blood pressure increased to 115/75 mmHg, SpO_2_ to 96%, whereas heart rate decreased to 75 beats/min. After 45 min in the operating theater, the patient regained consciousness, began to spontaneously breathe, and obeyed commands. The endotracheal tube was removed and the patient completely recovered.

## Clinical outcomes

**Figure 1:**
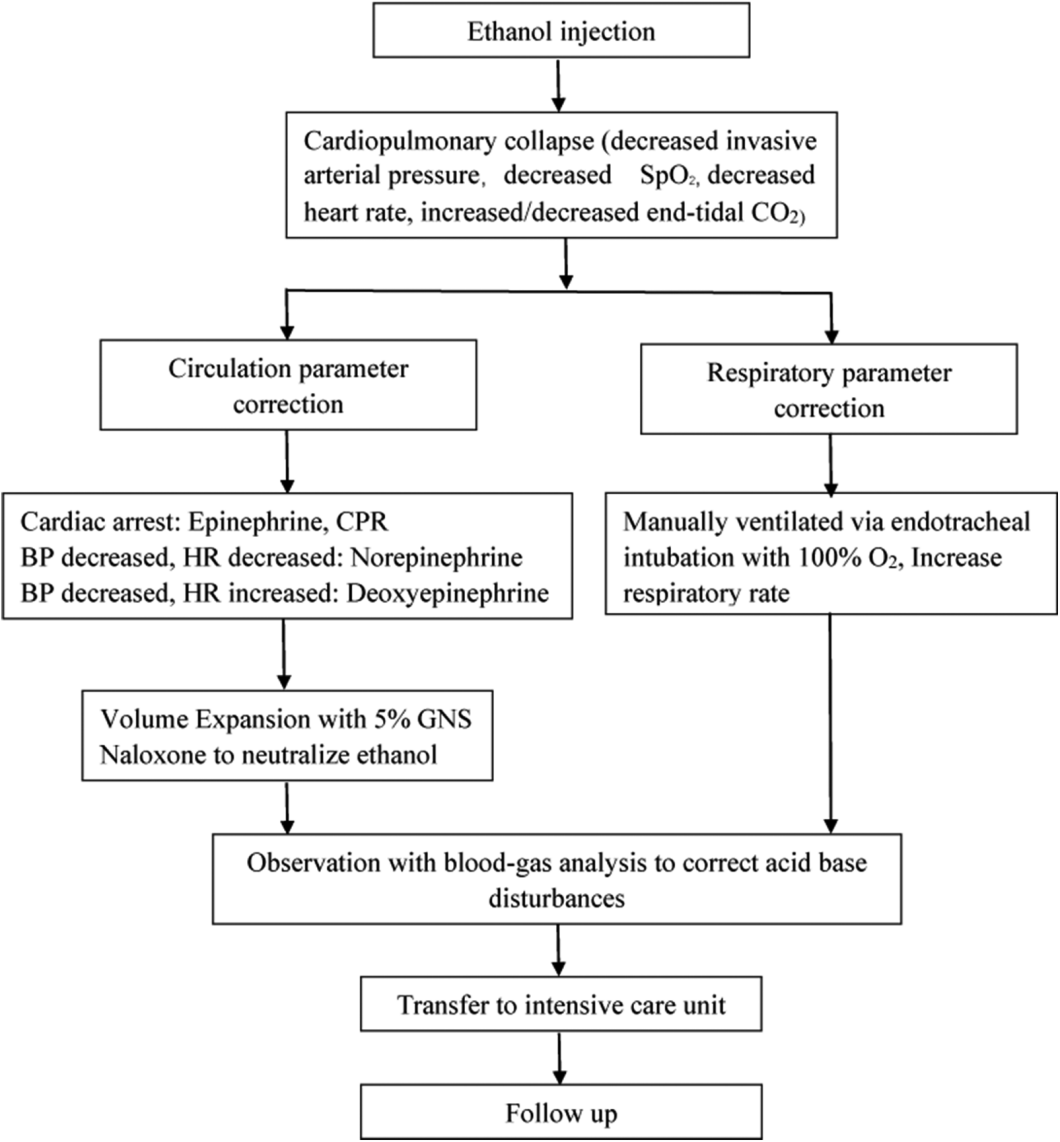
Steps in salvage of cardiopulmonary collapse induced by ethanol sclerotherapy for vascular malformations.

All three cases were successfully salvaged without major complications. When cardiopulmonary collapse occurred, manual ventilation via endotracheal intubation with 100% O_2_, increased ventilation frequency and external cardiac compression were the emergency treatments. Epinephrine, norepinephrine, deoxyepinephrine infusion solely or combined were crucial to maintaining the basic vital signs. The clinical data and results of 3 Patients with cardiopulmonary collapse were summarized in Table 1. The steps in salvage of cardiopulmonary collapse induced by ethanol sclerotherapy for vascular malformations was described in Figure 1.

**Table 1:** Clinical data and results of salvage of 3 patients with cardiopulmonary collapse.

Patient No.	Sex	Age (year)	Height (cm)	Weight (Kg)	Ethanol (ml)	Cardiac arrest	Epinephrine	Norepinephrine	Deoxyepinephrine	Recover time (min)	Complications
1	Female	32	166	58	22	Yes	1.0 mg	20 μ g/2 min × 3	10 μg/2 min × 3	50	No
2	Male	28	175	68	25	No	No	20 μg/2 min × 3	10 μ g/2 min × 2	35	No
3	Female	12	152	46	20	No	No	12 μg × 1	8 μg/2 min × 2	45	No

## Discussion

Numerous sclerosants have been developed for the treatment of vascular malformations. Among these, abso­lute ethanol is preferred to other embolic agents due to its relatively desired outcomes and minimal rate of recanalization. The injected ethanol in vascular malformations causes direct tissue toxicity leading to endothelial damage, severe vascular spasm, proteins denudation. Despite its benefits, ethanol sclerotherapy often produces minor local complications, such as tissue necro­sis, peripheral nerve injuries, skin blistering, and ulcers. Besides, if absorbed into the systemic circulation, although rare, ethanol may cause fatal complications, including pulmonary embolism and pulmonary va­sospasm. Because of changes in the cardiac conduction system, these complications may trigger right heart failure, cardiac arrhythmias, and even death in severe cases (7–9, 11, 12).

Systemic contamination with ethanol occurring during percutaneous sclerotherapy is directly related to the dose injected and independent of the vascular malformation morphology, venous drainage, or injection technique (13, 14). Theoretically, with the fast flow rate of arteriovenous malformations, absolute ethanol enters the systemic circulation more easily through the fistula compared to that of venous malformations, thereby increasing the incidence of cardiopulmonary accidents. This phenomenon has not been reported in clinical practice. All three cases in this work were venous malformations. The reasons were speculated as follows: First, doctors were more cautious about the speed of absolute ethanol injection in the treatment of arteriovenous malformations due to its high flow characters. Secondly, dominant outflow vein embolization with coils or compression was often used to control the flow speed of arteriovenous malformations. The rate of absolute ethanol entering the systemic circulation was highly decreased. Thirdly, doctors experienced in using absolute ethanol to treat vascular malformations should observe the fast flow rate of arteriovenous malformations. Absolute ethanol is more likely to be washed into the systemic circulation and diluted during the injection of absolute ethanol into arteriovenous malformations. it is not as easy to produce coagulation and hemolysis when treated venous malformations. Thus, micro-thrombosis and hemolysis may have less stimulation to pulmonary vessels.

Although the theory of pulmonary hypertension is not fully clear, acute pulmonary hypertension causes cardiopulmonary collapse. Based on previous studies, ethanol injection induces severe pulmonary vasospasm, acutely increases thin-walled right ventricular afterload, and decreases the right ventricular cardiac output. Besides, these effects can be further aggravated by systemic effects of alcohol affecting right atrial and ventricular contractility, including chronotropic and inotropic functions (15, 16). Pulmonary emboli due to ethanol-induced thrombosis may have also contributed but appeared less likely considering the rapid response of the patient to resuscitation. Ethanol and blood interaction produces embolic debris comprising denatured protein and cellular fragments described as “Sludge” by Yata (17). The thrombus produced by absolute ethanol injection is mostly micro-embolism, which will not produce pulmonary embolism as severe as deep venous thrombosis of the lower extremity. Nevertheless, the influence of micro-embolism may be cumulative. This may be the reason there is a dose limit in ethanol injection therapy.

Furthermore, ethanol-induced hemolysis releases erythrocyte arginase, which hydrolyses L-arginine to L-ornithine. Notably, L-Arginine is the precursor for nitric oxide (NO) production via NO synthase in the vascular endothelium. The combination of decreased production and increased inactivation of NO tips the vascular balance toward vasoconstriction (18). Kielstein et al. investigated the relationship between percutaneous ethanol injection, pulmonary hypertension, and markers of altered NO metabolism. Specifically, ethanol, free hemoglobin, plasma nitrite, and L-arginine levels were analyzed before and after percutaneous ethanol injection treatment (19). NO is effective in both primary pulmonary hypertension and secondary pulmonary hypertension (SPH), of which ethanol-induced type is SPH (20). In Case #1, our anesthesiologist used 68% NO. The use of NO in anesthesia uses the secondary gas effect to increase the alveolar concentration of inhaled anesthetics and thus deepen the depth of anesthesia as quickly as possible. At the same time, NO can dilate pulmonary vessels and reduce pulmonary vascular resistance, so as to reduce pulmonary artery pressure, but has no effect on systemic pressure. However, if the concentration of NO in the inhaled gas is too high, it may lead to insufficient oxygen inhalation resulting in hypoxia, so the concentration of NO used clinically does not exceed 60%–70%. When rescuing patients, NO inhalation will be stopped, and 100% oxygen inhalation will be changed to improve the inhaled oxygen concentration and increase oxygen supply.

NO has little to zero effect on the peripheral vasculature and is very pulmonary specific so is the perfect agent in that cardiopulmonary collapse critical clinical situation and is administered quickly to act through the in-dwelling endotracheal tube.

In cardiopulmonary collapse the circulation is very sluggish (and may have zero cardiac output) and the circulatory system is severely compromised due to the significantly decreased left-heart filling and right heart overload. Also it could be stated that if any IV drugs are considered for vasodilation, the immediate placement of a catheter in the main Pulmonary Artery (PA) to administer drugs IV into the lungs would then facilitate drug placement to get where it was needed more expeditiously. However, placing a catheter in the main PA does take time to do and is a delay, whereas endotracheal NO is immediate without any additional catheter procedures. Further, placing a catheter in the main pulmonary artery can aggravate the cardiac conduction system and invoke an aggravating arrhythmia further complicating and adversely affecting this already critical situation.

Ethanol injection procedures are performed when the patient is intubated and under general anesthesia for numerous reasons, including severe pain during injection, the need for cessation of respiration during angiographic sequences, and the possibility of cardiovascular decompensation. Also, alcohol severely affects the heart. Studies have reported cardiopulmonary collapse among adults during ethanol sclerotherapy presumably related to the suppression of the myocardial conduction system with precapillary vasoconstriction and resultant elevation in pulmonary arterial pressures (21–24). In the present three cases, we observed an increase in airway resistance, a decrease in blood SpO_2,_ and an increase in end-tidal carbon dioxide during the early stages of cardiopulmonary collapse. This implies that pulmonary factors may cause impairment of cardiopulmonary circulation. The proposed physiologic sequence for cardiovascular collapse as following steps: (1) A bolus of ethanol flows into the pulmonary vasculature. (2) If enough of a bolus, it causes diffuse severe pulmonary artery spasm. (3) This then restricts right heart outflow to the left heart, leading to right heart overload, then right heart failure. (4) This then results in decreased left heart filling with oxygenated blood, leading to blood pressure dropping. (5) Significantly decreased left heart filling and collapsing blood pressure, leads to coronary artery hypoperfusion, then cardiac ischemia, and then resultant arrhythmias, usually Electro-Mechanical Dissociation (EMD) which is organized electrical depolarization of the heart without synchronous myocardial fiber shortening (no myocardial contractures), and then there is no cardiac output. A similar phenomenon was also reported by Naik (6).

Invasive arterial pressure monitor provides accurate, reliable, and continuous arterial pressure value, and establishes the status of the blood volume or the heart. The slight increase in blood pressure among patients during ethanol sclerotherapy related to pain caused by sympathetic stimulation, even when patients are under general anesthesia. For the prevention of cardiopulmonary accidents, the observation of the decrease of invasive arterial pressure has more clinical significance. A rapid decrease in blood pressure indicates the possibility of cardiopulmonary impairment. This should be timely corrected to avoid cardiac arrest accompanied by a further decrease in blood pressure.

In the ethanol injection cases monitored using the Swan-Ganz catheter, nimodipine is administered once the pulmonary artery pressure has increased. This can quickly relieve the pulmonary artery pressure (25). Due to the high cost and the technique reason, Swan-Ganz catheter was not regularly be used by absolute ethanol sclerotherapy, especially for low flow vascular malformation. In our study, the three patients had a significant decrease in blood SpO_2_, peripheral blood pressure, and heart rate. Thus, the mechanisms of immediately improving the ventilation exchange function and maintaining the stability of basic vital signs are vital for cardiopulmonary resuscitation. Absolute ethanol injection was immediately stopped once blood SpO_2_ decreases and airway resistance increases. It should be noted that patients can be apneic during general anesthesia and minutes can pass before O_2_ levels drop. The critical factor to monitor is CO_2_ levels which are the very first to drop way before O_2_ levels drop. CO_2_ level is the first measurable parameter to drop physiologically because it measures the oxygen-carbon dioxide exchange at the alveolus and capillaries. O_2_ goes into the red blood cells to oxygenate the blood. CO_2_ is excreted into the alveolus and exhaled. The CO_2_ monitor reveals the CO_2_ level excreted. If there is no oxygen exchange occurring at the alveolus, there is no CO_2_ released for excretion and for measurement. Thus, once CO_2_ levels begin to fall, action must be taken then, not when O_2_ levels later drop which may be harder to correct. Besides, the anesthetic agents were discontinued and the patient was placed under 100% O_2_ and manually ventilated via endotracheal intubation to prevent further deterioration of cardiopulmonary impairment. Laryngeal Mask Airway was not recommended for general anesthesia for ethanol injection, which could not provide sufficient pressure when necessary.

Several types of drugs are used to increase blood pressure, including epinephrine, norepinephrine, deoxyepinephrine, dopamine, etc. Each drug has a different pharmacological effect and mechanism thus, requiring the guidance of professional anesthesiologists and physicians. Epinephrine is primarily used for the treatment of patients with acute allergy and cardiac arrest. On the other hand, norepinephrine is majorly used for the emergency treatment of patients with severe blood pressure reduction and heart rate reduction. Deoxyepinephrine is primarily used to raise blood pressure. Unlike norepinephrine, the effect of deoxyepinephrine in increasing heart rate is softer and used for blood pressure maintenance after the recovery of basic vital signs. A reasonable combination of various pressure-raising drugs to maintain the basic vital signs was crucial to the successful management of cardiopulmonary accidents caused by absolute ethanol sclerotherapy.

## Conclusion

In conclusion, we reported three patients successfully salvaged from cardiovascular collapse potentially attributed to ethanol sclerotherapy. Despite its severity, the resulting complications can be reversed with timely treatment, which indicated our salvage techniques were effective in the adult and pediatric age groups. We recommend that anesthetists should continuously focus on the changes of airway pressure, SpO_2_, tidal carbon dioxide, and invasive arterial pressure during the procedure and in the recovery room to prevent severe cardiovascular complications.

## Data Availability

The original contributions presented in the study are included in the article/Supplementary Material, further inquiries can be directed to the corresponding author.
